# HPV burden in Armenia among unvaccinated women: a series of cross-sectional population-based prevalence surveys

**DOI:** 10.1016/j.vaccine.2025.127405

**Published:** 2025-08-30

**Authors:** Iacopo Baussano, Vanessa Tenet, Karina Baghdasarova, Zaruhi Harutyunyan, Alex Vorsters, Daniëlle Heideman, Maaike Bleeker, Ricardo Rüttimann, Gayane Sahakyan

**Affiliations:** a International Agency for Research on Cancer (IARC/WHO), Early Detection, Prevention and Infections Branch, Lyon, France; b FIDEC (Fighting Infectious Diseases in Emerging Countries), Erevan, Armenia; c The National Center of Oncology after V.A. Fanarjyan, Yerevan, Armenia; d Centre for the Evaluation of Vaccination, Vaccine & Infectious Disease Institute, Faculty of Medicine and Health Sciences, University of Antwerp, Antwerp, Belgium; e Department of Pathology and Medical Biology, University Medical Center Groningen, University of Groningen, Hanzeplein 1, 9713, GZ, Groningen, The Netherlands; f Amsterdam UMC, location Vrije Universiteit Amsterdam, Pathology, De Boelelaan 1117, Amsterdam, The Netherlands; g FIDEC, Miami, USA; h National Center for Disease Control and Prevention of Ministry of Health, Armenia

**Keywords:** Human papillomavirus, Prevalence, Cervical cancer, Vaccination

## Abstract

**Background:**

In 2017, Armenia introduced a national human papillomavirus (HPV) vaccination programme with a quadrivalent vaccine at age 14 years. Successful implementation of the programme was affected by social media campaigns aiming to discredit its efficacy and safety, the COVID-19 pandemic, and local armed conflicts. To support national public health stakeholders, we initiated a series of studies to provide local evidence on the burden of HPV infection.

**Methods:**

Two cross-sectional HPV prevalence surveys among unvaccinated birth-cohorts of women were conducted: a urine-based survey (UBS) targeted women aged 17–21 years, and a cell-based survey (CBS) targeted women aged 21–39 years. In addition, we collected a series of invasive cervical cancer (CC) case laboratory samples to assess the attributable proportion of high-risk (HR) HPV types to estimate the impact of HPV vaccination in Armenia.

**Findings:**

In the UBS and the CBS, 2485 and 3017 women were included, respectively. In the UBS, 110 (4.5 %) women were positive for any HPV type, 72 (2.9 %) of which were HR HPV and 29 (1.2 %) were HPV16/18. In the CBS, 553 (18 %) were positive for any HPV type, 326 (11 %) of which were HR HPV, and 99 (3.3 %) were HPV16/18. In the CC series, HPV16/18 accounted for 71 % of all HR HPV infections, HPV 31, 33, 45, 52, and 58 accounted for an extra 18 %. The remaining HR types accounted for 11 % of all CC infected by HR HPV. The corresponding predicted cumulative 10-year CC incidence among 20–24, 25–34, and 35–44-year age group, was 0.3 %, 1.3 %, and 3.8 %, respectively.

**Interpretation:**

Our findings provide a picture of the HPV infection and future cervical cancer burden among unvaccinated young and adult women in urban areas of Armenia and can inform context-specific vaccination and screening policies.

## 1 Introduction

According to GLOBOCAN estimates, Cervical Cancer (CC) in Armenia has an age-standardised incidence rate of 6.8 cases per 100,000 person years (crude incidence rate 9.9) [1]. CC is preventable through both HPV vaccination and screening. Early detection and adequate treatment of invasive cancer considerably reduces related mortality. Indeed, vaccination against human papillomavirus (HPV) (with at least 90 % coverage), screening with a high-performance test (with at least 70 % coverage), and adequate treatment of at least 90 % of pre-cancerous lesions and invasive cancer are the pillars of the elimination initiative launched by the World Health Organization [2].

In 2017, Armenia introduced a facility-based national HPV vaccination programme with a quadrivalent (HPV16/18/6/11) vaccine on a two-dose schedule to girls aged 14 years [3]. Armenia's national HPV vaccination programme was targeted by social media campaigns aiming to discredit its efficacy and safety. It was also impacted the COVID-19 pandemic, as well as local armed conflicts. Nevertheless the programme was maintained throughout by the local public health authorities and full vaccination coverage (girls fully vaccinated by age 15 years) increased between 2018 and 2023 from 2 % to 30 % [3].

To sustain HPV vaccination and monitor its impact on CC, the Armenian Ministry of Health, in collaboration with the International Agency for Research on Cancer (IARC/WHO) and Fighting Infectious Diseases in Emerging Countries (FIDEC), initiated a series of studies to provide of local evidence on the burden of HPV infection. In 2019, as there was no data on the HPV burden among women in Armenia, we initiated two HPV prevalence surveys among unvaccinated birth cohorts and collected case series of invasive CC samples to provide information on the HPV burden in Armenia.

## 2 Methods

### 2.1 Study overview

Two separate cross-sectional HPV prevalence surveys among unvaccinated birth-cohorts of women were conducted in urban centres of Armenia. The first survey, conducted between June 2019 and September 2019, was based on the collection and testing of urine samples from young women irrespective of their sexual activity status and is hereafter referred to as a urine-based survey (UBS). The second survey, conducted between September 2021 and June 2022, was based on cervical samples collected from sexually active women and is hereafter referred to as a cell-based survey (CBS). For both surveys, study participants were asked to complete a short online risk-factor questionnaire. Since the full impact of vaccination on CC can only be seen decades after the introduction of HPV vaccination [4], to estimate the impact sooner, we also collected a series of invasive CC cancer case samples to assess the proportion of HR HPV types attributable to the burden. For this purpose, we retrieved 200 paraffin-embedded formalin-fixed blocks of invasive CC samples diagnosed between 2017 and 2019 from centralized pathology archives in Armenia. Further details about the population, procedures, and statistical analyses for each study are provided below.

### 2.2 Study population

For the UBS, we aimed to obtain an age-stratified sample of 2500 young women aged between 17 and 20 years at the time of recruitment. The target number of women to be recruited in each age group and recruitment centre was proportional to the number of women of that age resident in the catchment areas of the recruitment centres. A similar approach was adopted for the CBS, in which we aimed to recruit 2500 women aged from 21 to 25 years, our main target, and 500 women aged from 26 to 39, our secondary target.

Women residing in the cities of Yerevan, the capital, Abovyan, Ijevan and Armavir were invited to participate at the largest local health centres, either primary health-care or medical centres (see Table S1 in the appendix). Using health centre records, lists of potentially eligible young women in the appropriate age range were compiled for each centre. Eligible women were then invited to participate by phone (randomly) and during health centre visits for other reasons, or through their social networks (at their convenience). Women invited at the UBS were contacted through paediatric outpatient services of the primary health-care or medical centres, whereas those invited for the CBS were contacted through gynaecological outpatient services of the centres.

The following eligibility criteria were assessed before study enrolment: A) inclusion criteria, 1. being within the pre-defined age range at time of enrolment, 2. being healthy without obvious medical conditions according to medical history, 3. being able to understand and sign the written informed consent (IC) form, 4. being able to provide a biological sample and complete the anonymous web-based questionnaire, 5. being sexually active (only for the CBS); B) exclusion criteria, 1. having previously received HPV vaccination, 2. having any mental disability, 3. having undergone a hysterectomy, 4. any subject who – in the opinion of the investigator – was unlikely to comply with the age-appropriate sampling procedures described in the protocol, 5. failing to supply either the required urine or cervical sample, or to complete the anonymous web-based questionnaire.

Eligible women willing to participate to the surveys were asked to sign an IC form, which was recorded in the study registry. They were also asked to provide a biological sample, and to complete a short online questionnaire. Each participant was identified by a study identification number, which was also used to label the study registry, the IC form, the urine sample and the online questionnaire. This approach ensured the de-identification of the participants and the pseudo-anonymization of the collected information. Study Participants were guided through each step by trained local health-care personnel and were reassured about the confidentiality of the study.

### 2.3 Study procedures

#### 2.3.1 Urine and cervical sample collection

For the UBS, study participants were supplied with a device (Colli Pee™, Novosanis) designed to immediately dilute the first 14 ml of first-void urine into 7 ml of a urine-conservation medium to avoid DNA degradation. Collected urine samples were then transported from health-care facilities to the reference laboratory of the Armenian National Center for Disease Control and Prevention, where they were stored at −20 °C and shipped on dry ice to be tested. DNA extraction was performed as described elsewhere [5] at the Centre for the Evaluation of Vaccination, University of Antwerp. In the CBS, cervical cells were collected by a clinician in a ThinPrep medium, transported to the reference laboratory of the National Center for Disease Control and Prevention, and stored at 4 °C. All cervical cell samples were subsequently transferred for DNA extraction to Amsterdam UMC, at Vrije Universiteit, Amsterdam, the Netherlands. HPV DNA detection and genotyping of all samples was performed at Amsterdam UMC (as detailed below).

#### 2.3.2 Questionnaire collection

For both surveys, study participants were asked to complete a short online risk-factor questionnaire to assess the role of key risk factors, such as age, place of recruitment, place of birth, address, socio-economic level, and sexual behaviour. Digital data collected through the online questionnaire were transferred to IARC servers through a secured internet connection via local available infrastructures or local mobile data networks.

Local medical personnel underwent standardised training on the WHO guidelines for epidemiological studies [6], to learn how to invite participants, obtain IC forms, and collect biological samples and questionnaires. Compliance with CIOMS-WHO International Ethical Guidelines for Epidemiological Studies was confirmed by a Contract Research Organization (CRO).

It should be noted that this information was exclusively for study purposes. Study participants – as they were under the age of 30 years – did not receive their HPV test results. Indeed, as described in the recently published IARC handbook for cervical cancer screening, HPV infections in young women, in particular below age 30 years, clear rapidly, with 80 % to 90 % clearing within the two years [7]. Therefore, detection of HPV infection in urine of women under 30 does not have any specific clinical relevance. HPV-positive study participants under 30 years were not referred for further investigation. However, study participants who were aged 30 years or older were managed according to the local screening guidelines.

#### 2.3.3 Collection of CC biopsies/tissues and testing

A minimum of 200 formalin-fixed paraffin-embedded (FFPE) blocks of invasive cervical cancer samples diagnosed between 2017 and 2019 were retrospectively gathered from centralized pathology archives in Armenia. HPV DNA testing and genotyping, and histological confirmation of the CC diagnosis was performed, as described below, at Amsterdam UMC. H&*E*-guided core punch biopsies from the tumour area in the FFPE-blocks were taken and DNA was isolated as described previously [8]. For histologically-confirmed CC cases that were HR-HPV negative by GP5+/6+ PCR, extracted DNAs were additionally tested at IARC, Lyon, France, using a type-specific E7 PCR bead-based multiplex genotyping assay (E7-MPG) [9]. We complemented histology and sample genotyping with the following information completed by the local pathologist: year of diagnosis, age at diagnosis, histological subtype at diagnosis, and cancer staging according to the FIGO classification.

### 2.4 HPV detection and genotyping

HPV DNA detection and genotyping for all samples was performed at Amsterdam UMC, at Vrije Universiteit, Amsterdam, the Netherlands, using a general primer GP5+/6 + −mediated PCR. This was followed by hybridization of PCR products in an enzyme immunoassay using two oligoprobe cocktails to detect 13 HR HPV genotypes (HPV16, 18, 31, 33, 35, 39, 45, 51, 52, 56, 58, 59 and 68) and 29 low-risk (LR) HPV genotypes, respectively. Subsequent HPV genotyping was conducted using a microsphere bead-based assay (Luminex) or reverse line blot assay. Beta-globin PCR analysis was used to assess the quality of the DNA to be submitted to HPV PCR.

### 2.5 Statistical analyses

For both surveys, age- and type-specific HPV prevalence was estimated according to the methods adopted in previous HPV prevalence surveys conducted by IARC [10,11]. Specifically, HPV-DNA prevalence with the corresponding 95 % confidence intervals (CIs) and HPV type-specific prevalence in HR-HPV-positive invasive CC were both assessed assuming a binomial probability distribution.

Prevalence ratios (PR) for HPV-DNA positivity and the corresponding 95 % CIs for selected risk factors as measured using the questionnaires were estimated using a binomial regression model with a log link. PR estimates were adjusted by age group (≤17; 18; 19; and ≥20 years in the urine-based survey and ≤21; 22; 23; 24; 25; 26–28; 29–31; 32–34; and ≥35 in the cell-based survey). Risk trends were assessed by considering categories as continuous variables.

Finally, based on age-specific HPV prevalence and average age at sexual debut estimated through these surveys, using a validated statistical predictive model [12], we estimated age-specific cervical cancer incidence (along with corresponding 90 % prediction intervals [PIs]) after 10 years of follow-up in the birth cohorts aged 20–49 years at the time of the survey. Briefly, the predictive model (called “PANDORA”) was parameterized by fitting a Poisson regression model to assess the association between age-specific HR HPV prevalence (as measured in HPV prevalence surveys methodologically like those presented in this paper) and CC incidence (as measured through high-quality population-based cancer registries) in 17 different locations worldwide. Internal and external validity was also formally assessed, and model-based predictions could reproduce observed independent sets of data not used for the fitting process [12].

## 3 Results

### 3.1 Cross-sectional surveys

In the UBS, 3194 eligible women were invited through paediatric and family doctor services from 10 health centres, of which 2500 participated and 2485 aged 16–21 years were included (participation rate = 78.3 %). Eighty-eight percent of the young women were invited by phone, the rest were invited either while visiting the health centre or accompanying friends or relatives on visits (7 % and 5 % of the total, respectively). Most women were aged 17–20 years, except 3 who were aged 21 (see Table 1). Sixty-one percent of women recruited in the UBS were born in the capital, and 34 % were born elsewhere. Approximately 5 % were born abroad. Most (95 %) were living with family/relatives and were still at school (83 %), with a median duration of 12 years education. Eleven percent reported being professionally active, the rest were housewives or unemployed. The majority, (91 %), of women enrolled in the UBS reported no history of sexual intercourse, 94 % were never married (82 % of which had never had a boyfriend). Among 200 (8 %) women who reported being sexually active, the median age at sexual debut was 18 years.

**Table 1:** Description of women recruited for the urine-based survey, conducted in 2019 among women aged 17–20 years, and the cell-based survey, conducted 2021/22 among women aged 21–39 years in Armenia, by selected characteristics.

	**Urine-based survey**		**Cell-based survey**
**Characteristic**	**N (%)**	**Characteristic**	**N (%)**
**All**	2446	**All**	3017
			
**Medical centre**		**Medical centre**	
Yerevan Polyclinic #8	197 (8.1)	Yerevan Polyclinic #8	412 (13.7)
Yerevan Polyclinic #12	99 (4.1)	Yerevan Polyclinic #12	263 (8.7)
Yerevan Polyclinic #17	186 (7.6)	Yerevan Polyclinic #15	103 (3.4)
Yerevan Polyclinic #22	100 (4.1)	Yerevan Polyclinic #17	290 (9.6)
Armenia Republican MC	320 (13.1)	Surb Gr.Lusavorich MC	746 (24.7)
Surb Astvacamayr MC	275 (11.2)	Ijevan PHC	349 (11.6)
Surb Gr.Lusavorich MC	522 (21.3)	Abovyan MC	243 (8.1)
Ijevan PHC	257 (10.5)	Armavir MC	464 (15.4)
Abovyan MC	257 (10.5)	Karmir Blur Polyclinic	147 (4.9)
Armavir MC	233 (9.5)		
			
**Age-group (years)**		**Age-group (years)**	
≤17	690 (28.2)	≤21	193 (6.4)
18	575 (23.5)	22	224 (7.4)
19	585 (23.9)	23	445 (14.7)
≥20	696 (24.4)	24	560 (18.6)
		25	651 (21.6)
		26–28	248 (8.2)
		29–31	234 (7.8)
		32–34	228 (7.6)
		≥35	234 (7.8)
			
**Type of recruitment**		**Type of recruitment**	
By phone invitation	2163 (88.4)	By phone invitation	2155 (71.4)
While attending the polyclinic	168 (6.9)	Woman came opportunistically	691 (22.9)
Through friends/neighbor/relatives	113 (4.6)	Friends/neigbor recruited the woman	169 (5.6)
Other	2 (0.1)	Other	2 (0.1)
			
**Place of birth**		**Place of birth**	
Yerevan	1496 (61.2)	Yerevan	1244 (41.2)
Armenia, outside Yerevan	834 (34.1)	Armenia, outside Yerevan	1709 (56.7)
Abroad	116 (4.7)	Abroad	64 (2.1)
			
**Occupation**		**Annual household income in Armenian drams**	
Pupil	366 (15.0)	Less than 100,000	340 (11.3)
Student	1667 (68.1)	101,000–200,000	1144 (37.9)
Housewife/unemployed	157 (6.4)	201,000–300,000	846 (28.0)
Worker	256 (10.5)	301,000–400,000	314 (10.4)
		401,000–500,000	96 (3.2)
		More than 500,000	45 (1.5)
		Difficult to answer	232 (7.7)
			
**Number of years of school**		**Highest level of education**	
1–11	517 (21.1)	Incomplete secondary school	53 (1.8)
12	863 (35.3)	Complete secondary school	927 (30.7)
13–18	1066 (43.6)	Middle vocational educatio	763 (25.3)
Median (min-max)	12 (1–18)	Incomplete higher school	176 (5.8)
		Complete higher school	1080 (35.8)
		Postgraduate	18 (0.6)
			
**Place of living**			
With family/relative	2320 (94.9)		
With husband/cohabiting partner	107 (4.4)		
Alone/with friends	17 (0.7)	**Current marital status**	
Other	2 (0.1)	Never	67 (2.2)
		Married/living as married	2849 (94.4)
**Married**		Widowed	17 (0.6)
No	2296 (93.9)	Separated/divorced	84 (2.8)
Yes	150 (6.1)		
		**Marriages number**^1^	
**Ever had a boyfriend**^2^		1	2919 (98.9)
No	1880 (81.9)	2	30 (1.0)
Yes	381 (16.6)	3	0 (0.0)
Difficult to answer	35 (1.5)	4+	1 (0.03)
			
**History of sexual intercourse**		**Number of lifetime sexual partner**	
Never	2230 (91.2)	1	2904 (96.2)
Ever	200 (8.2)	2	83 (2.8)
Prefer not to answer	16 (0.7)	3	15 (0.5)
		4+	8 (0.3)
		Difficult to answer	7 (0.2)
			
**Age at first sexual intercourse**^3^**(yrs)**		**Age at first sexual intercourse (yrs)**	
≤ 17	36 (18.0)	≤ 19	1005 (33.3)
18	72 (36.0)	20–22	1320 (43.8)
≥ 19	79 (39.5)	≥ 23	686 (22.7)
Difficult to answer	11 (5.5)	Difficult to answer	6 (0.2)
Missing	2 (1.0)	Median (min-max)	20 (14–38)
Median (min-max)	18 (15–20)		
		**Age of the partner at first sexual intercourse (yrs)**	
		≤ 23	779 (25.8)
		24–27	1379 (45.7)
		≥ 28	845 (28.0)
		Difficult to answer	14 (0.5)
		Median (min-max)	25 (16–56)
			
		**Number of sexual partners in the last year**	
		0	103 (3.4)
		1	2882 (95.5)
		2	20 (0.7)
		3	0 (0.0)
		4+	1 (0.03)
		Difficult to answer	11 (0.4)
			
		**Current partner had sex before**	
		Yes	406 (13.5)
		Probably yes	737 (24.4)
		No	1107 (36.7)
		Don't know	767 (25.4)
			
		**Current partner had sex after**	
		Yes	75 (2.5)
		Probably yes	149 (4.9)
		No	2248 (74.5)
		Don't know	545 (18.1)
			
		**Age difference at first sexual intercourse (yrs)**	
		< 0	40 (1.3)
		0	245 (8.1)
		1–4 5–9 10	1193 (39.5)
		5–9	1204 (39.9)
		≥ 10	319 (10.6)
		Difficult to answer	16 (0.5)
			
		**Ever received any cash/kind for sex**	
		Never	2995 (99.3)
		Ever	13 (0.4)
		Difficult to answer	9 (0.3)
			
		**Pregnancy number**	
		0	529 (17.5)
		1	894 (29.6)
		2	870 (28.8)
		3	385 (12.8)
		4+	337 (11.2)
		Unknown	2 (0.1)
			
		**Age at first pregnancy**^4^	
		≤19	643 (25.8)
		20–21	784 (31.5)
		≥ 22	1061 (42.6)
		Median (min-max)	21 (14–39)
			
		**Ever have a PAP test**	
		Never	2410 (79.9)
		Ever	578 (19.2)
		Don't know	29 (1.0)

1 Among 2950 ever married women.

2 Among 2296 not married women.

3 Among 200 sexually active girls.

4 Among 2488 ever pregnant women.

In the CBS, 3017 women aged 21–39 years, were recruited through gynaecological services from nine centres, seven of which also participated in the UBS. For the CBS, we were not able to estimate the participation rate across all centres, for those with good quality data it ranged between 56 % to 85 %. Most women were recruited through phone invitation (71 %) or while attending the health centre for other medical reasons (23 %), i.e., opportunistically. A large majority of recruited women were born in Armenia (91 %), mostly outside the capital Yerevan (57 %). More than 90 % of recruited women reported being educated, 36 % completed secondary school, 25 % completed middle vocational education, and 36 % completed higher education, while two-third reported an annual household income between 101,000 and 300,000 Armenian Dram. Almost all women interviewed (98 %) had ever been married, mostly once (99 %), and 83 % of them had ever been pregnant, with a median age of first pregnancy at age 21 years. Women in the CBS reported a median age at first sexual intercourse of 20 years with a partner 5 years older (median age of 25 years), 96 % of women reported one lifetime sexual partner and almost none (>99 %) reported having ever received any cash or payment in kind for sex. Finally, 80 % of women interviewed reported never having a PAP smear. As expected, this percentage decreased with age (90 % or more among women younger than 25 years, compared to 26 % among women aged 35–39 years). Detailed flow charts (Appendix, Fig. S1) report the number of women included in the surveys.

**Fig. 1:**
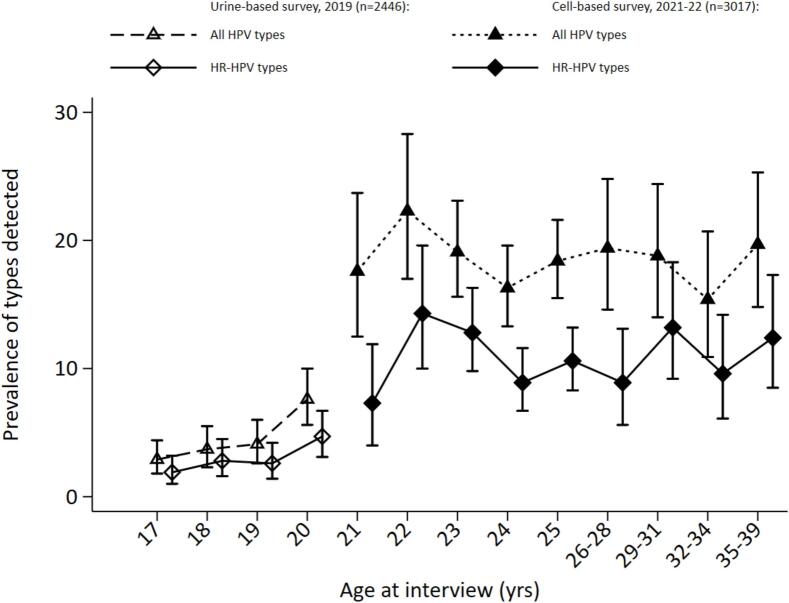
HPV prevalence by age.

In the UBS, 110 (4.5 %) women were positive for any HPV type, compared to 553 (18.3 %) in the CBS. In the UBS, 72 (2.9 %) were positive for a HR HPV infection compared to 326 (11 %) in the CBS. A detailed type-specific distribution of HPV infections, dominated by HPV16 and 31 in both surveys, is provided in the appendix, see Table S2. In table S3, we also show the age-specific distribution of HPV16 and 18 among recruited women. Fig. 1 shows the age-specific prevalence of all HPV types detected by GP5+/6+ test and HR HPV types in both the UBS and the CBS. In the UBS, the prevalence of all types and HR types, measured among all participants irrespective of sexual activity status, increased from 2.9 (1.8–4.4 95 %CIs) and 1.9 (1.0–3.2 95 %CIs) to 7.6 (5.6–10.0 95 %CIs) and 4.7 (3.1–6.7 95 %CIs) between ages 17–20 years, respectively, whereas in the CBS, prevalence peaked between ages 22 years at 22.3 (17.0–28.3 95 %CIs) and 14.3 (10.0–19.6 95 %CIs), respectively and subsequently plateaued at about 19.1 (15.6–23.1) and 12.8 (9.8–16.3), respectively.

PR for HPV-DNA positivity and the corresponding 95 % CIs for selected risk factors in both the UBS and the CBS are shown in Table 2. For the UBS, overall HPV prevalence was not affected by health centre, type of recruitment, place of birth, and age at sexual debut, however, it did increase with age at recruitment, lack of education, and history of sexual intercourse or partnerships. Girls at school at the time of enrolment were less likely to be HPV positive. For the CBS, the HPV prevalence was not affected by age at enrolment, annual household income, age at first pregnancy, and PAP smear history. A higher prevalence of HPV infection was observed among women recruited through friends or neighbours, in Yerevan Polyclinic #15, women with incomplete education, women who were never married or married more than once, as well as those who had had more than one lifetime sexual partner, or two or more sexual partners in the last year, women with an increasing age difference between their first sexual partner, and women who reported that their partner had sex with other women (before or during the existing relationship). Finally, HPV prevalence decreased when the number of pregnancies increased.

**Table 2:** Prevalence ratios (PRs) for human papillomavirus (HPV) positivity and corresponding 95 % confidence intervals (CIs) according to selected characteristics among 2446 girls in Armenia 2019 and women aged 21–39 years among 3017 women in Armenia 2021–22.

		**Urine-based survey**			**Cell-based survey**		
**Characteristic**	**N (%)**	**HPV-positive**^1^**N (%)**	**Adjusted**^2^**PR (95 % IC)**	**Characteristic**	**N (%)**	**HPV-positive**^1^ **N (%)**	**Adjusted**^2^**PR (95 % IC)**
**All**	2446	110 **(**4.5**)**		**All**	3017	553 (18.3)	
							
**Medical centre**				**Medical centre**			
Yerevan Polyclinic #8	197	9 (4.6)	1	Yerevan Polyclinic #8	412	74 (18.0)	1
Yerevan Polyclinic #12	99	2 (2.0)	0.51 (0.11–2.31)	Yerevan Polyclinic #12	263	58 (22.1)	1.22 (0.90–1.66)
Yerevan Polyclinic #17	186	2 (1.1)	0.26 (0.06–1.18)	Yerevan Polyclinic #15	103	34 (33.0)	1.87 (1.32–2.65)
Yerevan Polyclinic #22	100	2 (2.0)	0.48 (0.11–2.19)	Yerevan Polyclinic #17	290	53 (18.3)	1.00 (0.73–1.38)
Armenia Republican MC	320	16 (5.0)	1.12 (0.51–2.48)	Surb Gr.Lusavorich MC	746	129 (17.3)	0.95 (0.73–1.23)
Surb Astvacamayr MC	275	21 (7.6)	1.80 (0.85–3.85)	Ijevan PHC	349	50 (14.3)	0.77 (0.55–1.10)
Surb Gr.Lusavorich MC	522	30 (5.8)	1.23 (0.59–2.54)	Abovyan MC	243	45 (18.5)	1.01 (0.72–1.41)
Ijevan PHC	257	9 (3.5)	0.85 (0.34–2.09)	Armavir MC	464	80 (17.2)	0.95 (0.71–1.26)
Abovyan MC	257	7 (2.7)	0.64 (0.24–1.69)	Karmir Blur Polyclinic	147	30 (20.4)	1.13 (0.77–1.64)
Armavir MC	233	12 (5.2)	1.17 (0.50–2.71)				
							
**Age-group (yrs)**				**Age-group (yrs)**			
≤17	690	20 (2.9)	1	≤21	193	34 (17.6)	1
18	575	21 (3.7)	1.26 (0.69–2.30)	22	224	50 (22.3)	1.27 (0.86–1.87)
19	585	24 (4.1)	1.42 (0.79–2.54)	23	445	85 (19.1)	1.08 (0.76–1.55)
≥20	596	45 (7.6)	2.61 (1.56–4.36)	24	560	91 (16.3)	0.92 (0.64–1.32)
*χ*^*2*^_*3 for trend*_			*p < 0.0001*	25	651	120 (18.4)	1.05 (0.74–1.48)
				26–28	248	48 (19.4)	1.10 (0.74–1.63)
				29–31	234	44 (18.8)	1.07 (0.71–1.60)
				32–34	228	35 (15.4)	0.87 (0.57–1.34)
				≥35	234	46 (19.7)	1.12 (0.75–1.67)
				*χ*^*2*^_*8 for trend*_			*p = 0.669*
							
**Type of recruitment**				**Type of recruitment**			
By phone invitation	2163	94 (4.4)	1	By phone invitation	2155	382 (17.7)	1
While attending the polyclinic	168	10 (6.0)	1.21 (0.64–2.28)	Woman came opportunistically	691	127 (18.4)	1.04 (0.87–1.24)
Through friends/neighbor/relatives	113	6 (5.1)	1.19 (0.53–2.65)	Friends/neighbor recruited the woman	169	43 (25.4)	1.45 (1.10–1.90)
Other	2	0 (0.0)	–	Other	2	1 (50.0)	–
							
**Place of birth**				**Place of birth**			
Yerevan	1496	65 (4.3)	1	Yerevan	1244	254 (20.4)	1
Armenia, outside Yerevan	834	40 (4.8)	1.13 (0.77–1.65)	Armenia, outside Yerevan	1709	286 (16.7)	0.81 (0.70–0.95)
Abroad	116	5 (4.3)	1.02 (0.42–2.49)	Abroad	64	13 (20.3)	1.00 (0.61–1.65)
							
**Occupation**				**Annual household income in Armenian drams**			
Pupil	366	6 (1.6)	0.49 (0.20–1.20)	Less than 100,000	340	63 (18.5)	0.97 (0.76–1.25)
Student	1667	59 (3.5)	1	101,000–200,000	1144	217 (19.0)	1
Housewife/unemployed	157	21 (13.4)	3.62 (2.24–5.84)	201,000–300,000	846	135 (16.0)	0.84 (0.69–1.03)
Worker	256	24 (9.4)	2.43 (1.52–3.88)	301,000–400,000	314	58 (18.5)	0.98 (0.75–1.27)
				401,000–500,000	96	21 (21.9)	1.13 (0.76–1.69)
				More than 500,000	45	7 (15.6)	0.82 (0.41–1.64)
				Difficult to answer	232	52 (22.4)	1.18 (0.90–1.54)
				*χ*^*2*^_*5 for trend*_^3^			*p = 0.324*
							
**Number of years of school**				**Highest level of education**			
1–11	517	28 (5.4)	3.19 (1.89–5.39)	Incomplete secondary school	53	16 (30.2)	1.69 (1.10–2.61)
12	863	41 (4.8)	2.10 (1.34–3.30)	Complete secondary school	927	165 (17.8)	1
13–18	1066	41 (3.9)	1	Middle vocational educatio	763	148 (19.4)	1.10 (0.90–1.34)
*χ*^*2*^_*2 for trend*_			*p <* *0.0001*	Incomplete higher school	176	35 (19.9)	1.12 (0.81–1.56)
				Complete higher school	1080	186 (17.2)	0.97 (0.80–1.18)
				Postgraduate	18	3 (16.7)	0.91 (0.32–2.59)
				*χ*^*2*^_*5 for trend*_			*p = 0.325*
							
**Place of living**							
With family/relative	2320	83 (3.6)	1				
With husband/cohabiting partner	107	25 (23.4)	5.38 (3.48–8.33)				
Alone/with friends	17	2 (11.8)	2.64 (0.70–9.97)				
Other	2	0 (0.0)	–				
							
**Married**				**Current marital status**			
No	2296	78 (3.4)	1	Never	67	23 (34.3)	1.91 (1.37–2.70)
Yes	150	32 (21.3)	6.28 (4.31–9.15)	Married/living as married	2849	509 (17.9)	1
				Widowed	17	2 (11.8)	0.68 (0.19–2.51)
**Boyfriend**^4^				Separated/divorced	84	19 (22.6)	1.25 (0.84–1.88)
No	1880	41 (2.2)	1				
Yes	381	35 (9.2)	3.86 (2.46–6.04)	**Marriages number**^5^			
Difficult to answer	35	2 (5.7)	2.45 (0.62–9.73)	1	2919	518 (17.8)	1
				2+	31	12 (38.7)	2.12 (1.34–3.34)
							
**History of sexual intercourse**				**Number of lifetime sexual partners**			
Never	2230	62 (2.8)	1	1	2904	509 (17.5)	1
Ever	200	44 (22.0)	7.65 (5.07–11.55)	2	83	31 (37.4)	2.13 (1.59–2.86)
Prefer not to answer	16	4 (25.0)	8.52 (3.46–20.96)	3	15	7 (46.7)	2.68 (1.54–4.64)
				4+	8	4 (50.0)	2.89 (1.46–5.76)
				Difficult to answer	7	2 (28.6)	1.63 (0.50–5.26)
				*χ*^*2*^_*3 for trend*_^3^			*p < 0.0001*
							
**Age at first sexual intercourse**^6^**(yrs)**				**Age at first sexual intercourse (yrs)**			
≤ 17	36	9 (25.0)	0.52 (0.19–1.41)	≤ 19	1005	172 (17.1)	1
18	72	15 (20.8)	0.79 (0.41–1.53)	20–22	1320	244 (18.5)	1.09 (0.91–1.30)
≥ 19	79	19 (24.1)	1	≥ 23	686	133 (19.4)	1.20 (0.96–1.49)
Difficult to answer	11	1 (9.1)	0.21 (0.03–1.44)	Difficult to answer	6	4 (66.7)	4.01 (2.17–7.39)
*χ*^*2*^_*2 for trend*_^3^			*p = 0.148*	*χ*^*2*^_*2 for trend*_^3^			*p = 0.121*
				**Age difference at first sexual intercourse (yrs)**			
				< 0	40	8 (20.0)	1.36 (0.68–2.70)
				0	245	36 (14.7)	1
				1–4	1193	216 (18.1)	1.23 (0.89–1.71)
				5–9	1204	206 (17.1)	1.16 (0.83–1.61)
				≥ 10	319	83 (26.0)	1.76 (1.23–2.52)
				Difficult to answer	16	4 (25.0)	–
				*χ*^*4*^_*2 for trend*_^3^			*p = 0.018*
							
				**Number of sexual partners in the last year**			
				0	103	18 (17.5)	0.96 (0.62–1.47)
				1	2882	526 (18.3)	1
				2+	21	8 (38.1)	2.08 (1.20–3.62)
				Difficult to answer	11	1 (9.1)	0.47 (0.07–3.03)
				*χ*^*2*^_*2 for trend*_^3^			*p = 0.223*
							
				**Current partner had sex before**			
				Yes	406	96 (23.7)	1.62 (1.29–2.03)
				Probably yes	737	146 (19.8)	1.36 (1.11–1.66)
				No	1107	163 (14.7)	1
				Don't know	767	148 (19.3)	1.32 (1.08–1.62)
							
				**Current partner had sex after**			
				Yes	75	21 (28.0)	1.66 (1.14–2.42)
				Probably yes	149	38 (25.5)	1.51 (1.13–2.02)
				No	2248	381 (16.95)	1
				Don't know	545	113 (20.7)	1.23 (1.02–1.48)
							
				**Ever received any cash/kind for sex**			
				Never	2995	549 (18.3)	1
				Ever	13	3 (23.1)	1.27 (0.47–3.45)
				Difficult to answer	9	1 (11.1)	0.63 (0.10–4.00)
							
				**Pregnancy number**^7^			
				0	529	133 (25.1)	1
				1	894	180 (20.1)	0.79 (0.65–0.96)
				2	870	126 (14.5)	0.55 (0.44–0.69)
				3	385	57 (14.8)	0.54 (0.40–0.72)
				4+	337	57 (16.9)	0.61 (0.45–0.82)
				unknown	2	0 (0.0)	–
				*χ*^*2*^_*4 for trend*_^3^			*p < 0.0001*
							
				**Ever have a PAP test**			
				Never	2410	449 (18.6)	1
				Ever	578	96 (16.6)	0.88 (0.69–1.11)
				Don't know	29	8 (27.6)	1.44 (0.79–2.63)

1 HPV positive among 44 types detected by GP5+/6+.

2 Adjusted for age as appropriated.

3 Difficult to answer or unknown are not included in this test for trend.

4 Among 2296 not married women.

5 Among 2950 married women.

6 Among 200 sexually active girls.

7 Among 2488 parous women.

**Fig. 2:**
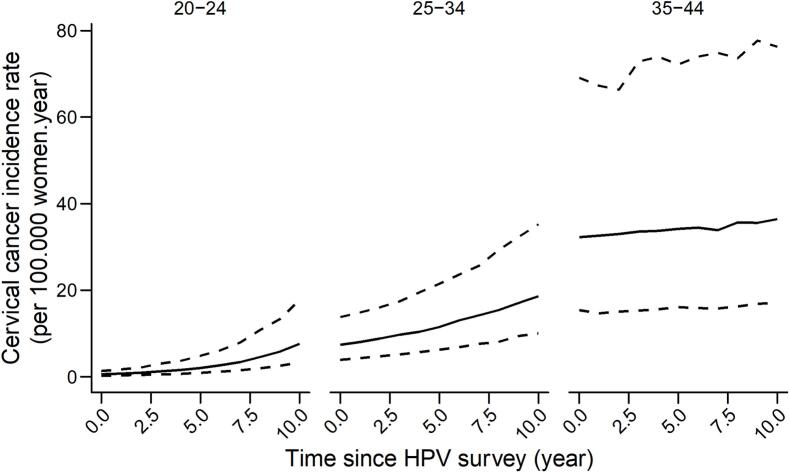
Estimated cervical cancer incidence among women recruited in the cell-based survey, using the PANDORA model.

We then used the age-specific HR HPV prevalence and the average age at sexual debut of women aged 20–24, 25–34, and 35–44 years in the CBS to project the expected 10-year CC incidence among these age groups in the following 10 years. Incidence, along with the relative 90 % prediction intervals, is shown in Fig. 2 and ranges between 0.6 and 7.6 per 100,000 women-year for the 20–24 year age group, between 7.4 and 18.6 for the 25–34 year age group, and between 32.3 and 36.4 for the 35–44 year age group. The predicted cumulative 10-year CC incidence was 0.3 %, 1.3 %, and 3.8 %, for each age group respectively.

In the case series, we included 206 confirmed invasive CC samples (see Fig. S2 in the appendix for the selection process) detected between 2017 and 2019; 51 % of which were diagnosed before age 55 years (see Table 3). Approximately 84 % of the blocks tested were obtained from biopsies, and 16 % were from surgical excisions. Ninety-two percent were squamous cell carcinomas, with 85 % of all cancers advanced in their progression, i.e., FIGO stage III or more. One or more HR HPV types were detected in 90 % of all cancers (see Table 4). HPV 16 and 18 accounted for 64 % of all cervical cancers and 71 % of all HR HPV infections (HPV 18 was more frequent in adeno(squamous)carcinomas). HPV 31, 33, 45, 52, and 58 accounted for an extra 16 %, and the remaining HR types accounted for 10 % of all cervical cancers.

**Table 3:** Description of the case series of invasive of cervical cancers, conducted in years 2017–19 in Armenia, by selected characteristics.

	**All cervical cancers**	
	N	%
**Total**	206	100

**Age at diagnosis**		
<45 years	45	21.8
45–54 years	60	29.1
≥55 years	101	49
Mean age (SD)	54.8 (11.8)	

**Year of Diagnosis**		
2017	30	14.6
2018	70	34
2019	106	51.4

**Tissue**		
Biopsy	172	83.5
Surgical incision	34	16.5

**Histological types**		
SCC	189	91.7
ADC/ADSC	17	8.3

**FIGO stage**		
I–II	31	15.1
III–IV	175	84.9

**Table 4:** Prevalence of HPV types among 206 cervical cancers, according to histological type.

	**SCC**		**ADC/ADSC**		**All**	
**HPV type**	**N**	**%**	**N**	**%**	**N**	**%**
Total	189	100	17	100	206	100

HR HPV negative	18	9.5	3	17.6	21	10.2
Any HR HPV	171	90.5	14	82.4	185	89.8
Multiple HR HPV	7	3.7	0	0.0	7	3.4

HPV16	99	52.4	5	29.4	104	50.5
HPV18	23	12.2	6	35.3	29	14.1
HPV45	15	7.9	1	5.9	16	7.8
HPV59	7	3.7	0	0.0	7	3.4
HPV31	6	3.2	1	5.9	7	3.4
HPV56	5	2.6	0	0.0	5	2.4
HPV58	5	2.6	0	0.0	5	2.4
HPV35	4	2.1	0	0.0	4	1.9
HPV52	4	2.1	0	0.0	4	1.9
HPV39	4	2.1	0	0.0	4	1.9
HPV68	2	1.1	1	5.9	3	1.5
HPV33	2	1.1	0	0.0	2	1.0
HPV51	2	1.1	0	0.0	2	1.0

Any alpha 7	28	14.8	2	11.8	30	14.6
Any alpha 9	21	11.1	1	5.9	22	10.7

HPV16/18	120	63.5	11	64.7	131	63.6
HPV31/33/45/52/58	31	16.4	2	11.8	33	16.0
HPV other HR	20	10.6	1	5.9	21	10.2

## 4 Discussion

Our findings provide a picture of the HPV infection burden among unvaccinated young and adult women in urban areas of Armenia. This information provides a baseline to monitor the impact of Armenia's HPV vaccination programme, and to project the future CC burden and evaluate the impact of screening. The present findings can be confidently compared with other IARC HPV surveys that were conducted according to similar sampling and HPV testing protocols [[13], [14], [15]]. [16]Age-standardised HR HPV prevalence among sexually active women aged 20 to 39 years in Armenia (10.8 %) is lower to rates, among women of the same age and birth cohorts, observed in Poland (16.0 %) in 2006 [17] and similar to neighbouring Georgia (9.8 %) in 2007 [18]. Overall, consistent with findings from previous IARC HPV surveys, we find that girls and women who are less educated – a proxy for earlier sexual activity – and who are more sexually active (or with a more sexually active partner) were at a higher risk of being HPV positive.

Furthermore, our projections for CC incidence in Armenia, among investigated birth-cohorts, are comparable to the annual CC incidence per 100,000 women reported in Georgia for women in the same age range. CC incidence rates among ages 30–34 years were 7.6 (3.3, 17.7) in Armenia versus 8.9 (6.5, 11.8) in Georgia. CC incidence rates among ages 35–44 years were 18.6 (10.6, 35.2) in Armenia versus 23.0 (20.1, 26.2) in Georgia. CC incidence rates among ages 45–54 years were 36.4 (17.1, 76.2) in Armenia versus 39.9 (36.1, 44.0) in Geogia [12]. Similarly, HPV16/18 were the most frequently detected HR types in the survey, ranging between 1.2 % and 3.3 % in young and adult women from the general population, up to 70.8 % among HR-HPV-positive ICC cases. In comparison, the proportion of ICC positive for the seven HR HPV types (HPV16/18/31/33/45/52/58) targeted in the existing nonavalent vaccine was 88.6 %. The proportion of HPV16/18 and HPV16/18/31/33/45/52/58 in ICC cases were in line with those estimated in Poland (79.5 % and 93.2 %, respectively) [17] and Georgia (68.1 % and 89 %, respectively) [18]. In a recent meta-analysis, the proportion of HPV16/18 and HPV16/18/31/33/45/52/58 detected in ICC was 79.9 % and 95.7 % respectively for Europe, and 83.2 % and 95.9 % respectively for Western/Central/South Asia [19]. In a previous study [20], based on similar proportions (72.5 % and 89.2 % respectively) from a systematic review [21], we estimated that the number of lifetime CC cases in Armenia expected in the absence of HPV vaccination among women born between 2005 and 2014 would be 1875 (95 % uncertainty intervals 1179-2983). In comparison, the expected maximum number of cancers prevented through HPV16/18 vaccination would be 1360 (95 % credible intervals 854-2172), and 1673 (1053-2658) would be prevented through HPV16/18/31/33/45/52/58 vaccination.

This study has weaknesses and strengths. As our surveys were conducted in urban settings, it is unclear to what extent our study population is representative of the women living in rural parts of Armenia. Some level of self-selection of study participants cannot be excluded, however participation rates were approximately 78 % in the UBS and 56–85 %, whenever these could be assessed, in the CBS, hence it is unlikely that self-selection has substantially distorted our estimates. A notable recruitment difference between the UBS and the CBS is that in the former survey, all young women were invited irrespective of their sexual activity status, whereas in the latter we only invited women who were ever sexually active. The choice to adopt different sampling methods with respect to previous sexual activity in the two surveys has been dictated by local cultural preferences and pragmatic reasons: in the UBS it would have been inconvenient and counterproductive to enquiry about previous sexual activity, since young women were invited through paediatric outpatient services, by contrast in the CBS women were invited through gynaecological outpatient services, in that case it was deemed acceptable by local health-care workers to invite only sexually active women. This must be accounted for when interpreting and comparing HPV prevalence estimates across the two surveys. For the UBS, given the relatively high participation rate, we do not expect there to have been any major distortion in our HPV prevalence estimates. For the CBS, conducted among adult and sexually active women, HPV prevalence among younger women may possibly have been overestimated. Indeed, according to the local Demographic Health Survey conducted in 2016, 26 % of women had never had sex at age 25 years. However, this proportion decreased to 11 % among women aged 30 years or more, therefore an overestimation is less likely as age of the women increases [22]. Furthermore, we have used self-collected first-void urine and clinician-collected cervical cells as biological samples for the UBS and CBS, respectively, however it has been previously shown that these sampling methods are comparable for the monitoring of HPV vaccine impact [23,24]. Strengths of our study include the large sample size of women, especially among younger women recruited, the availability of registries from which women were randomly invited and the supportive collaboration from doctors and nurses from the local health centres.

These two baseline surveys represent the first part of a long-term approach to monitoring HPV vaccination impact in Armenia. HPV prevalence surveys among women in the same age range in vaccinated birth cohorts will need to be repeated in the future. It is noteworthy that while only 15 % of the target population is fully vaccinated, the proportion of the population vaccinated with a single dose increased from 6 % in 2018 to 43 % in 2022 [3]. This coverage is far from being sufficient, but may still provide a detectable population-level impact, since a single-dose of quadrivalent HPV vaccination has been shown to effectively protect against HPV16/18 infection for at least a decade [4].

Currently, public health authorities in Armenia are re-organizing the national HPV vaccination programme to improve baseline coverage, mainly by addressing lack of information and re-engaging commitment from both health-care workers and civil society [25,26].

## 5 Conclusion

In conclusion, we have assessed the HPV burden among women under age 40 in Armenia and the proportion of ICC cases attributable to the different HR HPV types. We have also provided estimates of the corresponding expected burden of cervical cancer among these women. The above-reported findings can inform the design of context-specific vaccination and screening policies in Armenia.

## Abbreviations

**Table:** 

CBS	cell-based survey
CC	cervical cancer
CIs	confidence intervals
CRO	Contract Research Organization
E7-MPG	E7 PCR bead-based multiplex genotyping assay
FFPE	formalin-fixed paraffin-embedded
FIDEC	Fighting Infectious Diseases in Emerging Countries
HPV	human papillomavirus
HR	high-risk
IARC	International Agency for Research on Cancer
IC	informed consent
LR	low-risk
PR	Prevalence ratios
UBS	urine-based survey

## CRediT authorship contribution statement

**Iacopo Baussano:** Writing – review & editing, Writing – original draft, Visualization, Validation, Supervision, Resources, Methodology, Investigation, Funding acquisition, Conceptualization. **Vanessa Tenet:** Writing – review & editing, Methodology, Investigation, Formal analysis, Data curation, Conceptualization. **Karina** **Baghdasarova:** Writing – review & editing, Validation, Project administration, Investigation. **Zaruhi Harutyunyan:** Writing – review & editing, Validation, Investigation. **Alex Vorsters:** Investigation, Validation, Writing – review & editing. **Daniëlle Heideman:** Writing – review & editing, Validation, Investigation. **Maaike Bleeker:** Writing – review & editing, Validation, Investigation. **Ricardo Rüttimann:** Writing – review & editing, Resources, Project administration, Investigation, Funding acquisition, Conceptualization. **Gayane Sahakyan:** Writing – review & editing, Resources, Project administration, Methodology, Investigation, Conceptualization.

## Ethical approval

The present study had the approval of both the Ethics Committee of the Yerevan State Medical University (N4/2019) and the IARC Ethics Committee (Project No 18-29).

## Funding

The study was supported by the 10.13039/100000865Bill & Melinda Gates Foundation (Grant number: OPP-1188709). The funder had no role in the study design, data collection, data analysis, data interpretation, writing of the report or decision to submit for publication. The authors were not precluded from accessing data in the study, and they accept responsibility to submit for publication.

## Declaration of competing interest

The authors declare the following financial interests/personal relationships which may be considered as potential competing interests: IB reports financial support was provided by Bill & Melinda Gates Foundation. AV is a co-founder and former board member of Novosanis (subsidiary of OraSure Technologies Inc), Wijnegem, Belgium, a spin-off company of the University of Antwerp, and was a minority shareholder until January 2019. DH is minority shareholder of Self-screen B.V., a spin-off company of Amsterdam UMC, location VUmc, which develops, manufactures and licenses high-risk HPV and methylation marker assays for cervical cancer screening and holds patents on these tests. DH is currently employed at UMCG. The laboratory part of this study was performed during the time DH was employed at Amsterdam UMC. All other authors declare no competing interests. If there are other authors, they declare that they have no known competing financial interests or personal relationships that could have appeared to influence the work reported in this paper.

## Data Availability

External researchers can make written requests for sharing of data before publication or presentation. Requests will be assessed on a case by-case basis in consultation with lead and co-investigators.

## Appendix A Supplementary data

Supplementary material. Image 1
