# Supplementary material for: HPV burden in Armenia among unvaccinated women: a series of cross-sectional population-based prevalence surveys
Source: Vaccine. 2025 Aug 30;62:None. doi: 10.1016/j.vaccine.2025.127405 (PMC12447088; doi:10.1016/j.vaccine.2025.127405)
Supplement: Supplementary file 1 — Supplementary material. [file mmc1.pdf]

Appendix

Figure S1: Flow chart of the urine-based (left panel) and cell-based (right panel) surveys.

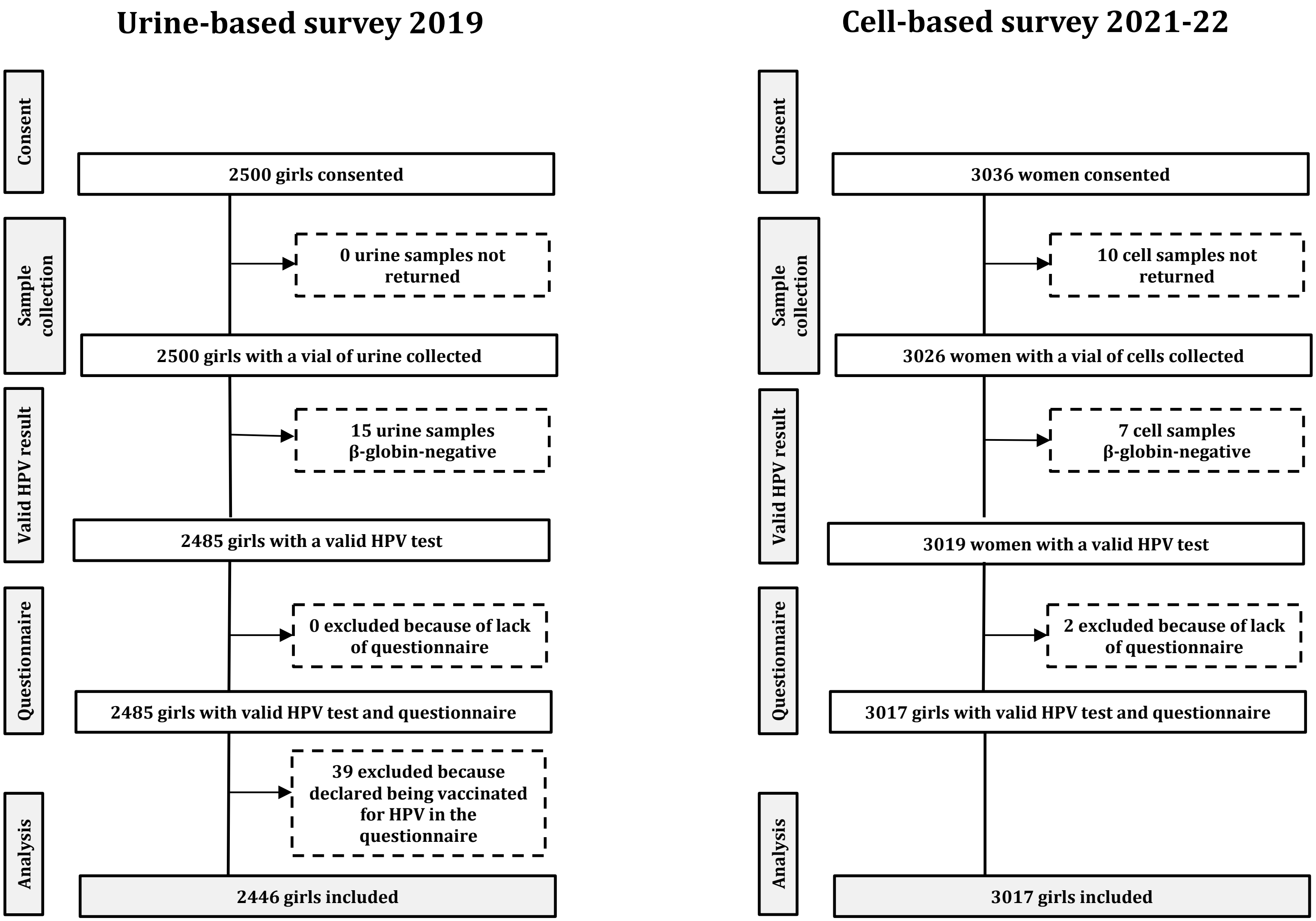

Figure S2: Flow chart of the invasive cervical cancer case series.

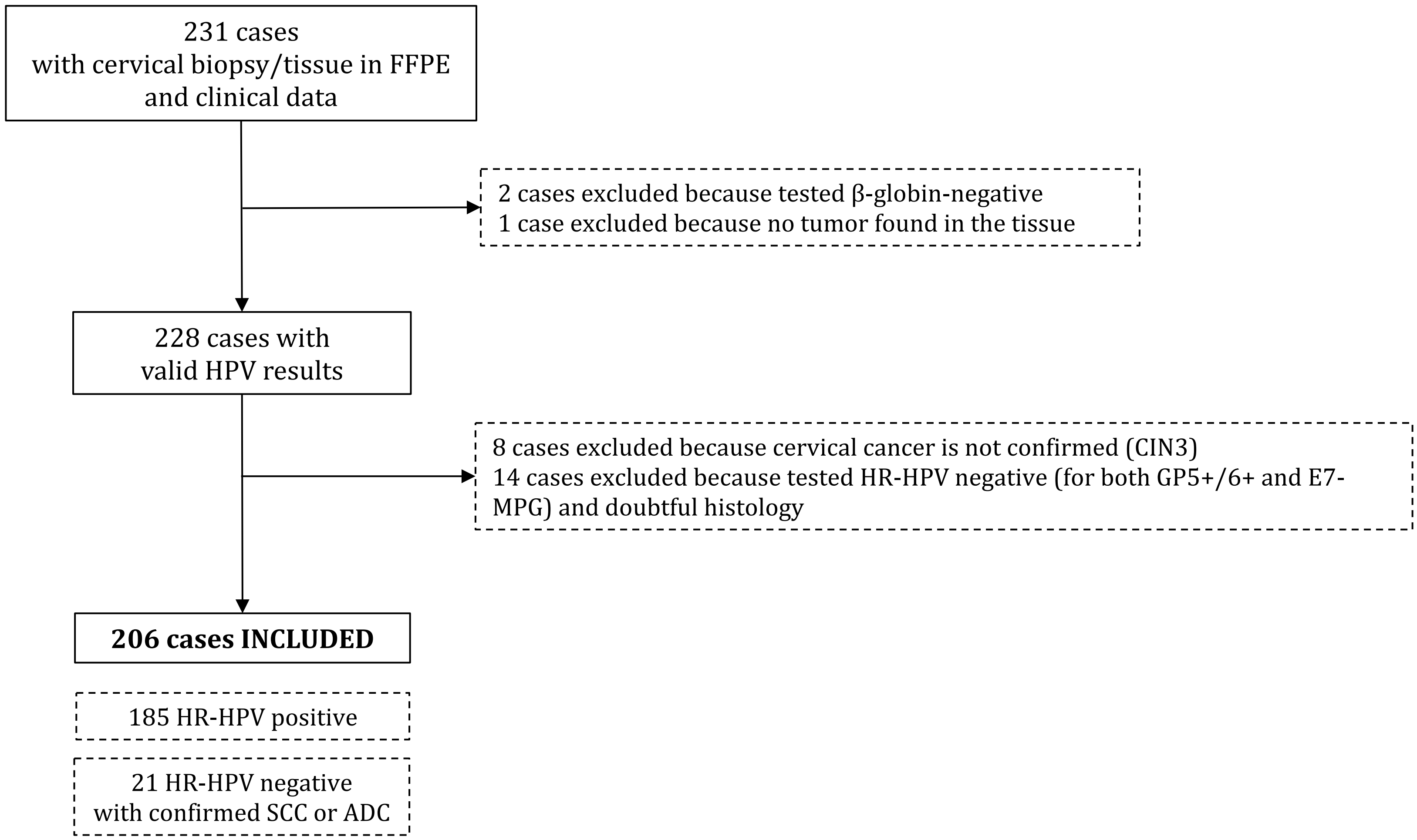

Table S1: List of local primary health-care (PHC) and medical centers (MC), and their potential eligible young women in the cities of Yerevan, Abovyan, Iyevan and Armavir, Armenia.

URINE-BASED SURVEY	NUMBER OF WOMEN BORN IN YEARS 1999 TO 2002[1]	CELL-BASED SURVEY SURVEY	NUMBER OF WOMEN BORN IN YEARS 1996 TO 2000[2]
01 = Yerevan Polyclinic #8	705	01 = Yerevan Polyclinic #8	910
02 = Yerevan Polyclinic #12	732	02 = Yerevan Polyclinic #12	849
03 = Yerevan Polyclinic #17	661	03 = Yerevan Polyclinic #15	847
04 = Yerevan Polyclinic #22	437	04 = Yerevan Polyclinic #17	900
05 = Armenia Republican MC	1249	05 = Surb Gr.Lusavorich MC	2935
06 = Surb Astvacamayr MC	968	06 = Ijevan PHC	1151
07 = Surb Gr.Lusavorich MC	2043	07 = Abovyan MC	1144
08 = Ijevan PHC	1031	08 = Armavir MC	1545
09 = Abovyan MC	851	09 = Karmir Blur Polyclinic	450
10 = Armavir MC	914		

[1]1999 to 2002 are the years of birth of women aged 17 to 20 in 2019.

[2]1996 to 2000 are the years of birth of women aged 21 to 25 in 2021-22.

Table S2. Prevalence of overall HPV types among 2446 girls aged 17-20 years in Armenia, 2019 and 3017 women aged 21-39 in Armenia, 2021-22. All HPV types detected by GP5+/6+.

HPV type	Urine-based survey (n=2446)				Cell-based survey (n=3017)			
	Single	Multiple	Total (%)		Single	Multiple	Total (%)	
**HPV-**	-	-	2336	95.5	-	-	2464	81.7
**HPV+**	63	47	110	4.5	393	160	553	18.3
High-risk								
**16**	4	13	17	0.7	47	33	80	2.7
**18**	5	7	12	0.5	7	13	20	0.7
**31**	7	13	20	0.8	40	23	63	2.1
**33**	1	3	4	0.2	9	10	19	0.6
**35**	0	2	2	0.1	1	4	5	0.2
**39**	0	1	1	0.0	7	11	18	0.6
**45**	2	1	3	0.1	7	12	19	0.6
**51**	0	6	6	0.2	9	18	27	0.9
**52**	1	6	7	0.3	6	4	10	0.3
**56**	4	7	11	0.4	12	13	25	0.8
**58**	0	5	5	0.2	13	9	22	0.7
**59**	3	7	10	0.4	11	17	28	0.9
**68**	1	1	2	0.1	0	3	3	0.1
**X**	2	0	2	0.1	33	2	35	1.2
**Any**	30	42	72	2.9	202	124	326	10.8
Low-risk								
**6**	1	3	4	0.2	2	14	16	0.5
**11**	0	2	2	0.1	7	2	9	0.3
**26**	0	0	0	0.0	0	0	0	0
**30**	0	0	0	0.0	11	1	12	0.4
**32**	0	0	0	0.0	8	12	20	0.7
**34**	0	0	0	0.0	0	0	0	0
**40**	1	2	3	0.1	1	3	4	0.1
**42**	8	9	17	0.7	19	22	41	1.4
**43**	0	5	5	0.2	7	6	13	0.4
**44**	0	0	0	0.0	0	0	0	0
**53**	1	2	3	0.1	5	5	10	0.3
**54**	1	2	3	0.1	5	3	8	0.3
**55**	0	1	1	0.0	3	1	4	0.1
**57**	0	0	0	0.0	0	0	0	0
**61**	0	0	0	0.0	0	0	0	0
**64**	0	0	0	0.0	0	0	0	0
**66**	6	9	15	0.6	25	23	48	1.6
**67**	0	5	5	0.2	8	13	21	0.7
**69**	0	0	0	0.0	0	0	0	0
**70**	0	2	2	0.1	10	12	22	0.7
**71**	0	0	0	0.0	0	0	0	0
**72**	0	0	0	0.0	1	0	1	0
**73**	1	4	5	0.2	7	13	20	0.7
**81**	0	3	3	0.1	13	12	25	0.8
**82**	0	4	4	0.2	5	2	7	0.2
**83**	2	2	4	0.2	2	8	10	0.3
**84**	1	1	2	0.1	3	3	6	0.2
**85**	0	0	0	0.0	0	0	0	0
**86**	1	1	2	0.1	1	1	2	0.1
**89**	2	2	4	0.2	5	3	8	0.3
**90**	2	4	6	0.2	13	21	34	1.1
**X**	6	3	9	0.4	30	22	52	1.7
**Any**	63	47	70	2.9	191	139	330	10.9
**16/18**	9	20	29	1.2	54	45	99	3.3
**16/18/31/33/45/52/58**	20	36	56	2.4	129	87	216	7.2

Table S3. Prevalence by age of HPV 16 and 18 types among 2446 girls aged 17-20 years in Armenia, 2019 and 3017 women aged 21-39 years in Armenia 2021-22. HPV types detected by GP5+/6+.

Age	Total	HPV 16/18		
		N	(%)	CI 95%
17	690	6	0.9	0.3-1.9
18	575	8	1.4	0.6-2.7
19	585	6	1.0	0.4-2.2
20	596	9	1.5	0.7-2.8
21	193	8	4.1	1.8-8.0
22	224	6	2.7	1.0-5.7
23	445	21	4.7	2.9-7.1
24	560	18	3.2	1.9-5.0
25	651	20	3.1	1.9-4.7
26-28	248	8	3.2	1.4-6.3
29-31	234	7	3.0	1.2-6.1
32-34	228	5	2.2	0.7-5.0
35-39	234	6	2.6	0.9-5.5
